# Establishing a Robust Manufacturing Platform for Recombinant Veterinary Vaccines: An Adenovirus-Vector Vaccine to Control Newcastle Disease Virus Infections of Poultry in Sub-Saharan Africa

**DOI:** 10.3390/vaccines8020338

**Published:** 2020-06-26

**Authors:** Omar Farnós, Esayas Gelaye, Khaled Trabelsi, Alice Bernier, Kumar Subramani, Héla Kallel, Martha Yami, Amine A. Kamen

**Affiliations:** 1 Viral Vectors and Vaccines Bioprocessing Group, Department of Bioengineering, McGill University, Montreal, QC H3A 0G4, Canada; omar.farnosvillar@mcgill.ca (O.F.); alice.bernier@mcgill.ca (A.B.); kumar.subramani@mcgill.ca (K.S.); 2 Research and Development Department, National Veterinary Institute, P.O. Box 19 Debre Zeit, Ethiopia; esayasgelaye@gmail.com (E.G.); martha.yami@nvi.gov.et (M.Y.); 3 Group of Biotechnology Development, Institut Pasteur de Tunis, 13, Place Pasteur, B.P. 74, 1002 Tunis, Tunisia; khaled.trabelsi@pasteur.tn (K.T.); hela.kallel@pasteur.tn (H.K.)

**Keywords:** Newcastle Disease virus, adenovirus vaccine, vaccine manufacturing, bioreactor production, HEK293, veterinary vaccination, vaccine production platform

## Abstract

Developing vaccine technology platforms to respond to pandemic threats or zoonotic diseases is a worldwide high priority. The risk of infectious diseases transmitted from wildlife and domestic animals to humans makes veterinary vaccination and animal health monitoring highly relevant for the deployment of public health global policies in the context of “one world, one health” principles. Sub-Saharan Africa is frequently impacted by outbreaks of poultry diseases such as avian influenza and Newcastle Disease (ND). Here, an adenovirus-vectored vaccine technology platform is proposed for rapid adaptation to ND or other avian viral threats in the region. Ethiopian isolates of the Newcastle Disease virus (NDV) were subjected to sequence and phylogenetic analyses, enabling the construction of antigenically matched vaccine candidates expressing the fusion (F) and hemagglutinin-neuraminidase (HN) proteins. A cost-effective vaccine production process was developed using HEK293 cells in suspension and serum-free medium. Productive infection in bioreactors (1–3 L) at 2 × 10^6^ cells/mL resulted in consistent infectious adenoviral vector titers of approximately 5–6 × 10^8^ TCID_50_/mL (approximately 10^11^VP/mL) in the harvest lysates. Groups of chickens were twice immunized with 1 × 10^10^ TCID_50_ of the vectors, and full protection against a lethal NDV challenge was provided by the vector expressing the F antigen. These results consolidate the basis for a streamlined and scalable-vectored vaccine manufacturing process for deployment in low- and medium-income countries.

## 1. Introduction

Newcastle Disease (ND) is considered one of the most critical diseases affecting poultry in sub-Saharan Africa. An important number of outbreaks per year are projected for some countries in the area, with a significant economical negative impact and deterioration of food security [1,2,3]. Immunization with inactivated or live-attenuated ND vaccines, although protective, has some disadvantages such as the production of vaccines using specific pathogen-free embryonated eggs and the possibility of virus shedding leading to disease in non-vaccinated animals [4,5]. Additionally, most of the currently available vaccines are prepared using Newcastle Disease virus (NDV) strains isolated decades ago. Pathogen-free chicken embryonated eggs are imported from countries outside Africa, which increases significantly their cost in the manufacturing process. Egg-derived vaccine production methods are also less efficient in their production capacity compared to cell culture technologies and cannot be easily adapted to rapid responses in case of emerging threats [6,7]. In the sub-Saharan African region, millions of chickens are farmed, with the majority being kept in villages and rural settings in scavenging production systems, with constrains of feed, management, and disease issues [8,9]. In these scenarios, ND has devastating effects on both the commercial farms and the village chickens [10].

All strains of Newcastle Disease virus (NDV) belong to the order *Mononegavirales*, family *Paramyxoviridae*, and genus *Avulavirus* and are contained in one serotype. They are also known as avian paramyxovirus serotype-1 (APMV-1) [11,12]. NDV is an enveloped virus, with a non-segmented genome, composed of single-stranded, negative sense RNA containing six genes that include two surface glycoproteins: the fusion (F) protein and hemagglutinin-neuraminidase (HN) protein. These are the viral neutralization proteins and the major protective antigens [13]. The HN protein is responsible for viral attachment to the host cell, and the F protein mediates fusion of the viral envelope with the cell membrane [14,15]. NDV isolates vary widely in their pathogenicity in chickens. On this basis, NDV strains are classified as highly virulent (velogenic), intermediate (mesogenic), or avirulent (lentogenic) [16]. The disease is caused by only the virulent strains of the virus and the clinical signs can include depression, ruffled feathers, open mouth breathing, hyperthermia, anorexia, listlessness, and hypothermia before death [12]. All strains are classified under a single serotype, but both antigenic and genetic diversities are evident among NDV strains [17]. On the basis of genome length and the sequence of the F protein gene, NDV strains have been classified into two major classes. The class I strains have mainly been isolated from wild birds and are generally avirulent; contrary to class II, where genotypes III–IX and XI–XVI are all virulent [17,18].

Taking into consideration the economic burden of Newcastle Disease and the nature of the current production methods of NDV vaccines in sub-Saharan Africa, novel and more efficient technologies are needed with the objective to produce alternative ND vaccine candidates and to contribute as well to the introduction and expansion of cell culture technologies for other viral poultry pathogens in the region. New types of vaccines could potentially protect chickens against NDV and could reduce the amount of virus shed by vaccinated birds, preventing bird-to-bird viral transmissions in vaccinated flocks.

Adenoviral vectors (Ads) are widely studied and have been extensively evaluated as recombinant vaccines. Ads have many advantages as vaccine delivery vectors, including “self adjuvanting” properties [19,20]. Ads can be manufactured in mammalian cell culture systems, most commonly using HEK293 cells that provide E1 protein in *trans* to allow viral replication. These production systems support high viral yields at relatively low production costs. Many clinical and preclinical studies have demonstrated that recombinant Ads are safe, and Ad-vectored vaccines can be easily generated based on efficient productions at high titer (10^11^ VP/mL) [21]. Recently, a conditional license for production and use in emergency situations of an adenovirus-vectored vaccine against foot and mouth disease in livestock was granted in the US [22,23], and adenovirus vaccination in humans has been practiced for decades [24].

In this work, we present the design, development, and production in bioreactors of genotype-matched adenoviral vectored variants of NDV vaccine candidates expressing the F and HN antigens. The recombinant vaccines were produced using the HEK293SF cell manufacturing platform and serum-free medium, with repeated productions undertaken at different scales in order to fine-tune and validate its development. The vaccine candidates were finally evaluated in the target animal species to demonstrate their immunogenicity and protective capacity. This is also the first report on an adenoviral-vectored vaccine manufacturing process against NDV.

## 2. Materials and Methods

### 2.1. Phylogenetic Analysis of African Strains of the Newcastle Disease Virus

Four NDV isolates were collected in Ethiopia between the years 2013 and 2018. They were designated according to the geographical area and year of sample collection as NDV/Haramaya/2013, NDV/Legetafo/2014, NDV/Addis Ababa/2017, and NDV/Debre zeit/2018. Phylogenetic and nucleotide sequence divergence analyses were conducted in order to characterize the four isolates. The phylogenetic tree construction involved 53 nucleotide sequences representing various NDV genotypes. Four of them were the isolates of the present study, three were strains used as live-attenuated or thermostable vaccines, and 46 sequences were available in the GenBank database. The tree (evolutionary history) was constructed using the neighbor-joining algorithm and the software MEGA v7 [25]. The statistical significance of the tree topology generated was evaluated by 1000 bootstrap resampling of the data.

#### 2.1.1. RNA Isolation, cDNA Synthesis, and Nucleotide Sequencing

The RNA was extracted from allantoic fluids obtained from laboratory-infected eggs using RNeasy^®^ Mini kit (QIAGEN, Austin, Texas, USA) following the manufacturer’s instructions. The first-strand cDNA was synthesized by using a SuperScript RT-PCR kit utilizing random hexamers and the iScript™ Select cDNA Synthesis kit (BioRad, Hercules, CA, USA). For genotyping, a 749-bp variable region (positions 4677 to 5425) of the fusion protein gene encompassing the cleavage site with reference to the sequence of NDV strain LaSota (GenBank accession number AF077761) was targeted for the RT-PCR assay. The corresponding F-gene segment was amplified using the primers 5′-GGAATTGTGGTAACAGGAGACAAAG-3′ and 5′-ATATTATTGAGGTTCCCGACTGAGG-3′. The PCR products were visualized in 0.8% agarose gels and directly sequenced. DNA sequencing was performed by the Sanger method, and the sequences were analyzed by BLAST alignments, comparing with representative NDV isolates retrieved from Genbank.

#### 2.1.2. Accession Numbers and Nucleotide Sequences for Gene Cloning

The fragment sequences of 749 bp of the fusion protein gene were submitted to Genbank, and their accession numbers are the following: NDV/Haramaya/2013 (MN909675), NDV/Legetafo/2014 (MN909676), NDV/Addis/Ababa/2017 (MN909677), and NDV/Debre/zeit/2018 (MN909678). The Debre zeit sequence was used as a reference in conjunction with the isolate APMV-1/Ethiopia/13VIR3936-27/2012 (KJ958914) for completion of the fusion (1662 bp) and hemagglutinin-neuraminidase (1737 bp) gene sequences that were used in the construction of the NDV adenoviral vaccine vectors.

### 2.2. Cells Lines and Culture Media

HEK293A adherent cells were used with transfection protocols aimed at the rescue of the adenoviral vectors as described thereafter. HEK293A cells were also used for the Median Tissue Culture Infectious Dose (TCID_50_) assay. For this assay, HEK293A cells were maintained in cell culture dishes in a humidified incubator at 5% CO_2_ and 37 °C in Dulbecco’s Modified Eagles Medium (DMEM) (Wisent, QC, Canada), supplemented with 10% Fetal Bovine Serum (FBS) (Gibco, Gaithersburg, MD, USA) without antibiotics. Cells were passaged twice a week, and detachment at confluence was conducted with Trypsin (MilliporeSigma, Oakville, ON, Canada). After centrifugation at 400× *g* for 5 min, the cells were resuspended in fresh medium and seeded at 1:10 dilution.

The HEK293SF cell line is derived from HEK293 cells which were adapted for culture in suspension and serum-free medium. HEK293SF (clone 293SF-3F6) suspension cells were derived from the corresponding Good Manufacturing Practices (GMP) master cell banks [26,27]. These cells were used for amplification of the rescued viruses, for primary stock preparation, and for vector production in bioreactors (at a scale of 1–3 L). HEK293SF cells were grown either in disposable polycarbonate vented-cap shake flasks (Corning, Tewksbury, MD, USA) or bioreactors. For maintenance, cells were passaged twice per week by diluting to 2.5 × 10^5^ viable cells per mL in fresh medium. They were grown either in HyClone HyCell TransFx-H medium (GE Healthcare, Chicago, IL, USA) or Xell AG HEK-GM medium (Xell AG, Bielefeld, Germany). Both are chemically defined, animal-component-free, and protein-free media, with no antibiotics added. HyCell TransFx-H was supplemented with 6 mM Gibco GlutaMAX Supplement (Fisher Scientific, Saint-Laurent, QC, Canada) and with 0.1% Kolliphor poloxamer 188 (MilliporeSigma, Oakville, ON, Canada). When grown in HEK-GM, they were supplemented with 6 mM Gibco GlutaMAX Supplement (Fisher Scientific, Saint-Laurent, QC, Canada).

Chicken fibroblast (DF-1) cells (ATCC CRL-12203^TM^) from *Gallus gallus* were used for transduction with the recombinant adenoviral vectors for corroboration of NDV antigen expression.

### 2.3. Adenovirus Vectors Design, Rescue, and Seed-Stock Generation

#### 2.3.1. Adenoviral Vectors Design

The sequences of the F- (1662 bp) and HN- (1737 bp) genes of Newcastle Disease virus were chemically synthesized (Genscript Biotech, Piscataway, NJ, USA) and cloned into the adenoviral transfer vectors pShuttle and pShuttle-Cytomegalovirus (CMV) for the generation of replication-deficient human adenoviruses type 5 (Agilent Technologies, Saint-Laurent, QC, Canada) following the directions of the supplier [28]. These vectors were transformed in the BJ5183 *E. coli* strain which contains the pAdEasy plasmid (Agilent technologies, Saint-Laurent, QC, Canada) carrying the adenoviral genome deleted for the E1 and E3 regions. Adenoviral vectors were obtained by recombination of the adenoviral genome and the NDV coding sequences. Each gene was individually inserted into expression cassettes that contained the following regulatory elements: the first cassette contained the human CMV early enhancer fused to the chicken β-actin promoter, followed by a chimeric intron of the chicken beta-actin gene, one EcoR I cloning site, and the rabbit beta globin polyA signal. This cassette, suitable for protein expression in avian cells, was modified from the pCAGGS plasmid and was inserted into the pShuttle transfer vector. The second cassette was contained within the pShuttle-CMV and consists of the human CMV promoter, followed by a multiple cloning site and the SV40 polyadenylation signal for transcription termination. The first cassette served to separately introduce the F- or HN-genes into the EcoRI site under the control of the chicken β-actin promoter. Each cassette was extracted with restriction endonucleases (KpnI/XhoI for the F-gene cassette and EcoRV/SalI for the HN-gene cassette) and inserted into the pShuttle vector multiple cloning site. The transfer vectors generated were designated pShuttle-F-βactin and pShuttle-HN-βactin. For the CMV expression plasmid, the F and HN fragments were cloned into the Kpn I/HindIII sites of the vector pShuttle-CMV to produce the pShuttle-F-CMV and pShuttle-HN-CMV, respectively. Afterwards, the fragment containing the human CMV promoter followed by the HN gene was amplified by PCR (Platinum SuperFi DNA Polymerase, Invitrogen, Waltham, MA, USA) with the primers 5′-CCCAAGCTTGGGGCGTTACATAACTTACGGTAAATGG-3′ and 5′-CCGATATCTACTACCGGCTAGACCTGGCTT-3′ to introduce HindIII/EcoRV sites (underlined) at its 5′ and 3′ ends, allowing its insertion downstream of the F-gene in the pShuttle-F-CMV. The SV40 polyadenylation signal, which was synthesized with restriction sites Not I/Hind III, was then inserted between the CMV-F fragment and the CMV-HN-polyA cassette, completing a bicistronic vector with two transcription units, oriented head-to-tail. This vector was designated as pShuttle-F-HN-CMV.

In parallel, the HN-gene was inserted into the Kpn I/Hind III restriction sites of the pAdTrack-CMV plasmid to produce the pAd-HN-CMV-GFP transfer vector that also expresses the Green Fluorescent Protein (GFP). One Ad vector expressing only GFP as a foreign antigen was also used for immunization experiments as a negative control.

The transfer vectors as well as the final plasmids carrying the foreign genes were verified by restriction endonuclease analysis (New England Biolabs, Whitby, ON, Canada) and sequencing. A final digestion step using Pac I enzyme allowed transfection of HEK293A cells for the rescue of adenoviral-assembled particles after 7 days in culture. Additionally, in vitro expression of NDV-encoded antigens was confirmed by transfection of HEK293A and DF-1 cell lines.

#### 2.3.2. Adenovirus Rescue

Primary viral stocks were prepared after transfection into the E1-complementing HEK293A cells. In preliminary experiments, two different transfection reagents were evaluated, GenJuice (MilliporeSigma, Oakville, ON, Canada) and Polyethylenimine (PEI) (Linear, MW 25,000, PolyScience, Warrington, PA, USA) at different mass ratios of DNA–reagent (1:3 and 1:6). Transfections were conducted in 6-well plates with the pAd-HN-GFP-CMV vector in order to evaluate the transfection efficiency through GFP expression measured by flow cytometry, using a Fortessa X-20 (BD Biosciences, BD Biosciences, Mississauga, ON, Canada) cytometer. The DNA–reagent ratio of 1:6 was selected for the transfection of the adenoviral vectors Pac I-linearized in T-25 flasks seeded at 0.7 × 10^6^ total cells until 80% confluence. The transfections were completed, and the flasks were left for several days with the addition of 2 mL of medium every 48 h. In the case of the adenoviruses carrying the GFP coding sequence, the cells were visualized under the fluorescence microscope for GFP plaques at 5–6 days post-transfection. After seven days, the cells were collected and lysed by three snap freeze/thaw cycles. The cell culture lysate was cleared from cellular debris by centrifugation at 6000× *g* for 10 min at 4 °C. This supernatant was used for subsequent virus amplifications. The cells used for amplification were similarly processed, and the supernatants for each clone were pooled and analyzed for total (VP/mL) and infectious viral particles (TCID_50_/mL). Primary adenovirus stocks for the different adenovirus vectors were prepared and stored at −80 °C. These seed-stocks were further assessed for infectivity and expression of the antigens in a sequence of quality control assays using HEK293SF and DF-1 cells. HEK293SF in suspension were infected in 6-well plates or 125 mL shake flasks. For expression and characterization of the recombinant antigens, the multiplicity of infection (MOI) used was 1. For adenovirus transduction of adherent DF-1 cells, a MOI of 5 was used.

### 2.4. Analytical Assays and Characterization of the Recombinant Adenoviruses

#### 2.4.1. Cell Count and Viability

Cell growth and viability were monitored by determining live cell density using 0.2% trypan blue exclusion dye (Thermo Fisher Scientific, Waltham, MA, USA) in a Vi-CELL-XR Cell Viability Analyzer (Beckman Coulter, Montréal, QC, Canada) or manually using a hemocytometer.

#### 2.4.2. Total Particle Quantitation of Adenovectors

Total particle concentration was determined by using an anion-exchange high-performance liquid chromatography system (Waters Alliance HPLC system, Milford, MA, USA) as described in [29]. In brief, the system consists of a separation module e2695 with a mobile phase degasser and an autosampler, a photodiode array detector model 2998 and a UNO Q polishing column (0.46 cm i.d. × 1 cm long). Monitoring was conducted at 260 and 280 nm. The running flow rate was 1 mL/min. The Alliance HPLC system runs under the EMPOWER 3 software. A standard curve was created using the Ad5 standard from ATCC (ATCC^®^ VR-1516) with a range of 1.0E + 10 to 1.0E + 11 VP/mL. Total viral particle concentrations were calculated in the lysate of HEK293SF-infected cells and in purified material obtained from CsCl gradients. The infectious viral particles/total viral particles (IVP/VP) concentration ratio was also calculated.

#### 2.4.3. Infectious Particle Titration

Infectious titer (IVP/mL) was performed using HEK293A in 96-well plates. HEK293A cells were seeded at 20,000 cells/well in DMEM supplemented with 2% FBS and allowed to grow for 24 h. On the second day, serial dilutions of the virus samples were prepared in fresh growth medium that was added to the plate in duplicates, using two plates per sample to be analyzed (16 wells per sample). The starting dilutions were 10^−3^ or 10^−4^ depending on the expected titer. Subsequent 1:10 dilutions were performed horizontally in the plate, keeping one last column with no virus as a negative control. After 7 days, the cells were assessed for the development of a cytopathic effect as observed by the inverted microscope. The TCID_50_/mL value was calculated according to the Spearman–Kärber method [30]. A reference control was run in every determination to ensure an acceptable reproducibility of the assay.

In the case of GFP expression, this was performed in adherent HEK293A with supernatant dilutions from 1:100 to 1:10,000 and allows readings of GFP-expressing cells in the range of 2 to 50% positive cells by flow cytometry. The experiments were conducted in duplicate. For the calculation of the IVP/cell, the following formulas were used:

IVPs/mL = % of FCs per well × dilution factor × number of cells per well/100 × sample volume per well(1)

Specific viral production (IVPs/cell) = Ad-HN-GFP-CMV titer/total cell density of the sample(2)

FC: fluorescent cells.

#### 2.4.4. Enzyme-Linked Immunosorbent Assays (ELISA) and Hemagglutination Inhibition Assay (HIA)

For the measurement of serum-specific IgG antibodies elicited against the F- antigen, an indirect ELISA (ID Screen^®^ Newcastle Disease Indirect, IDVet, Grabels, France) adapted to mice, using plates coated with the recombinant F protein, was performed following the manufacturer’s instructions and employing sera samples from mice blood collected as described. A goat anti-mouse IgG horseradish-peroxidase antibody was used as secondary antibody, and readings were performed at 450 nm. According to the assay, a signal to positive (S/P) ratio > 0.3 or titers over the cutoff of 993 suggest the generation of protective immunity against NDV. A similar ELISA from the same manufacturer (IDvet, ID Screen^®^ Newcastle Disease Indirect Conventional Vaccines, NDVS-CV-5P, France) was used to evaluate the presence of NDV-specific IgY antibodies in immunized chickens. Here, plates are coated with total NDV antigens instead of a single protein. The resultant optical density values were used to calculate the S/P ratio and the antibody titers of the samples as follows:

S/P = OD sample—OD negative control/OD positive control—OD negative control

Antibody titer:

log_10_(titer) = 1.00 × log_10_(S/P) + 3.520 (3)

Titer = 10^log10(titer)(4)

The hemagglutination inhibition assay (HIA) was used with mice or chicken sera to detect antibodies with hemagglutination inhibition (HI) activity as described previously [31]. The HI titer was identified as the highest serum dilution at which no hemagglutination was observed. HI titers are considered to be protective against NDV if they are above log_2_(3). Positive and negative control sera from chickens were included.

#### 2.4.5. PCR Analyses and Sequencing

For confirmation of the identity of the adenoviral vectors obtained, the High Pure Viral Nucleic Acid Kit (Roche, Mississauga, ON, Canada) was used for the extraction of high purity viral DNA from the culture broth of transfected HEK293SF cells after lysis and clarification by centrifugation. PCR reactions were run using the oligonucleotide primers summarized in Supplementary Material 1, which also provides the nucleotide sequences used for the construction of the vaccine vectors.

### 2.5. Cell Culture and Upstream Process Development for Adenoviral Vector Production

#### 2.5.1. Cell Growth Kinetics and Media Performances

Cell growth and viability of HEK293SF cells were compared using two commercial serum-free, chemically defined basal culture media. HyCellTransFx (GE Healthcare, Chicago, IL, USA) and HEK-GM (Xell AG, Bielefeld, Germany) were evaluated in 125 mL shake flasks with 25 mL of initial working volume maintained on an orbital shaker platform (Infor’s HT, Montréal, QC, Canada) at 110 revolutions per minute (rpm) in conditions of 80% humidity, 5% CO_2_, and 37 °C. Cultures were seeded at a cell density of 0.25 × 10^6^ cells/mL, and the cells were monitored daily. The addition of feed supplements was also assayed in identical cultures run in parallel, following the manufacturers’ recommendations. Supplementing the HEK-GM basal media consisted of a bolus addition of HEK-FS (Xell AG, Bielefeld, Germany) supplemented with 4 mM GlutaMAX™ (MilliporeSigma, Oakville, Ontario, Canada), starting from day 2 of the culture at 3% (v/v), then increasing to 4 and 5% (v/v), and then 10% (v/v) until the end of the culture. Supplementing the HyCellTransFx medium was carried out with Cell Boost™ 5 (GE Healthcare, Chicago, IL, USA, USA) every second day by adding a bolus at 5% (v/v) supplemented with 4 mM GlutaMAX™ (MilliporeSigma, Oakville, Ontario, Canada) until the end of the culture. Cell counts were performed daily.

#### 2.5.2. Virus Production at Small Scale

For infection and virus production characterization, experiments were conducted using the Ad-HN-CMV-GFP vector for quantification of infectious viral particles by measurements of GFP-expressing cells analyzed by flow cytometry (Fortessa X-20, BD Biosciences, Mississauga, ON, Canada). HEK293SF cells were cultured until reaching densities of 1, 2, 4, and 6 × 10^6^ cells/mL. Infection with the adenoviral virus stock was initiated at each of these cell densities at an MOI = 1. Throughout the production, cells were monitored daily for cell density, viability, and adenovirus production. For every shake flask analyzed, samples were collected up to 72 h postinfection (hpi) and the cells were lysed with three snap freeze/thaw cycles. The resulting lysed cell culture supernatants from the different flasks and different time points were analyzed for GFP expression in the TCID_50_/mL assay.

### 2.6. Bioreactors, Operating Conditions and Online Data Processing

Adenoviral production in 1 L bioreactors (Applikon Biotechnologies, Delft, The Netherlands) was first conducted in batch mode (working volume 0.75 L) with the Ad-F-CMV, Ad-HN-CMV, and Ad-F-HN-CMV vectors. Fed-batch productions of Ad-F-CMV, Ad-F-HN-CMV, and Ad-F-βactin were also completed in 1 L or 3 L controlled bioreactors (Applikon Biotechnology, Delft, The Netherlands,) with the objective to extensively document the production process and to generate material for downstream purification studies as well as for animal studies.

The 1 L bioreactor was equipped with a single marine impeller, a pH sensor, a temperature sensor, a dissolved oxygen (DO) concentration sensor, and a micro sparger with 100 um pore size.

The 3 L bioreactor (working volume 2.7 L) was equipped with a double marine impeller and a capacitance probe (Aber instruments Ltd., Aberystwyth, UK) to measure cellular biomass. The reactors were seeded at a viable cell density ranging between 0.38 to 0.66 × 10^6^ cells/mL in Xell AG HEK-GM medium. Cells were grown until the time of infection, at approximately 2 × 10^6^ cells/mL, and were infected using a MOI of 1. The culture runs were supplemented with a 5% bolus feed at a viable cell count of approximately 1 × 10^6^ cells/mL. The feeding was maintained as described. The bioreactor control unit ensured controlled conditions of DO concentration at 40% air saturation by continuous surface aeration of 5 or 12.5 mL/min (for the 1 or 3 L units, respectively) and injection of pure oxygen when required. The pH was set to 7.15 and regulated by the injection of CO_2_ into the headspace or the addition of NaHCO_3_ (90 g/L) (MilliporeSigma, Oakville, Ontario, Canada). Agitation was kept at 100 rpm and increased to 120 rpm in the last 24 h of culture in order to avoid the formation of cell aggregates. Monitoring of the culture characteristics, such as cell growth and the infection process, was conducted through the analysis of capacitance values (uF/cm) in the 3 L bioreactor as an indication of the total biovolume of the culture, using the capacitance probe. The bioreactor control unit, equipped with a proportional-integral-derivative (PID) controller suite, ensured controlled conditions of process parameters throughout the run. Cells were harvested when cell viability reached around 70–80%, generally between 48–60 hpi. Samples were taken every 24 h to measure cell density as well as to determine VP/mL by anion-exchange HPLC using absorbance at 260 nm as the detection method. The TCID_50_/mL (IVP/mL) was also determined, and the ratio IVP/VP was calculated.

### 2.7. Purification of the Recombinant Adenoviruses

#### 2.7.1. Cells Harvest, Lysis, and Free Nucleic Acid Digestion

At the time of harvest, the bulk of the cells were collected by centrifugation at 400× *g* for 15 min at 4 °C and resuspended in spent medium to 1/5th to 1/10th of the initial volume present in the shake flask or bioreactor. Cell lysis consisted of three snap freeze/thaw cycles, alternating between a dry ice/ethanol bath and 30 °C water bath. After lysis, the lysates were subjected to DNAse (Benzonase endonuclease, MilliporeSigma, Oakville, ON, Canada) treatment at 10 units per mL lysate for one hour at 37 °C with agitation and the addition of MgCl_2_ at 2 mM final concentration. The lysate was centrifuged at 6000× *g* for 15 min at 4 °C, and the cell debris discarded. The supernatant containing the released virus was aliquoted and frozen at −80 °C for further use. For the determination of virus production yields, analytical samples were taken from the bioreactors at the appropriate time points and frozen at −80 °C. On the day of the analysis, the aliquots were thawed at room temperature and then centrifuged at 4500× *g.* The cell culture lysate supernatants contained Ads accumulated in the cell culture supernatant and Ads released after cell lysis. They were analyzed for total and infectious particle concentration.

#### 2.7.2. Purification by CsCl Ultracentrifugation

By resuspending the cells in 1/5th- to 1/10th-fold less volume than the starting culture volume, we effectively concentrated the cells by 5- or 10-fold, thereby increasing the adenovirus concentration by the same factor. For purification by CsCl ultracentrifugation, 20 mL of this concentrated cell culture lysate supernatant was loaded onto a step CsCl gradient (8 mL in the bottom of a 53 g CsCl + 87 mL 10 mM Tris-HCl, pH 7.9, and 6 mL on top of a 26.8 g CsCl + 92 mL 10 mM Tris-HCl, pH 7.9, solution) and ultracentrifuged at 28,500 rpm (100,000× *g*) at 4 °C for 1 h and 30 min in a Beckman SW32 Ti rotor and using Ultra-Clear Beckman Centrifuge Tubes (25 × 89 mm). Two bands can be seen, and the lowest band (closest to the bottom of the tube) corresponds to intact viral particles. The virus band was collected by side-puncturing the tube using an 18 gauge needle. Dialysis was immediately carried out using the Slide-A-Lyzer™ G2 Dialysis Cassettes (300 kDa cutoff, 15 mL capacity) against 2 rounds of 2 L of formulation buffer (10 mM Tris, 100 mM NaCl, and 2 mM MgCl_2_ 2% sucrose pH 7.5 sterile-filtered). The purified recombinant adenoviruses were quantified and characterized by the anion-exchange HPLC and TCID_50_ assays.

### 2.8. Animals, Immunization Experiments, and Viral Challenge

#### 2.8.1. Ethics Statement

The studies involving experimentation with animals were in accordance with guidelines and recommendations from the Guide for the Care and Use of Laboratory Animals (Eighth Edition) and policies from the Institute Pasteur de Tunis, Tunisia, and from the National Veterinary Institute, Ethiopia. When mice or chickens were used for experimentation, they were housed in appropriate rooms with feeding, water supply, and health monitoring. The animals were euthanized humanely. The experimental protocols were drafted by the authors and approved by the respective Institutional Committees of Ethics.

#### 2.8.2. Vaccination and Challenge NDV Strains

The Newcastle disease virus isolate NDV/Debre zeit/2018 (MN909678) was used for in vitro Hemagglutination Inhibition Assays (HIA) in order to evaluate sera from mice and chicken immunized with the recombinant adenoviruses. It was also used for administration of an intramuscular challenge to chickens consisting of a lethal dose of 0.5 × 10^6.5^ ELD_50_. Live thermostable vaccine based on I2 strain was prepared at the National Veterinary Institute (NVI, Debre zeit, Ethiopia) and was used for the generation of hyperimmune sera in chickens to be used as a positive control of hemagglutination inhibition activity and to be injected as a positive control in an immunization and challenge experiment in chickens.

#### 2.8.3. Mice Experiment

A vaccination experiment was conducted in mice using the CMV adenoviral constructions in order to obtain evidence on the immunogenicity of the vectors. Different groups of randomly selected Balb/c mice (*n* = 8) seronegative to NDV were immunized with the Ad-F-CMV, Ad-HN-CMV, Ad-F-HN-CMV, and Ad-GFP-CMV (as negative control) adenoviruses. Two 200 μL doses at 1 × 10^7^ TCID_50_ were administered per animal 21 days apart via the intramuscular (i.m.) route. For the measurement of serum-specific IgG antibodies elicited against the NDV F antigen, serum samples were obtained from blood collected at days 0, 28, and 60 and stored at −80° C until analysis by ELISA.

#### 2.8.4. Target animal experiment

Two-week old male and female chickens were randomly selected and segregated into five groups; each one composed of 10 chickens which were reared in-house at NVI and maintained under standard conditions with ad libitum feed and water. The experimental groups were immunized twice at 1 × 10^10^ TCID_50_ with the recombinant adenoviruses Ad-F-CMV, Ad-F-HN-CMV, and Ad-F-βactin without any adjuvant at 300 μL/dose in addition to the NDV live vaccine which was also injected via intramuscular as directed. A fifth group of non-immunized animals was included. Immunizations were administered on days 0 and 21. Blood samples were collected on days 14, 28, and 35 for serological analysis. At day 56 of the experiment, all chickens were intramuscularly challenged with the NDV isolate Debre zeit/2018 (MN909678) at a lethal dose of 0.5 × 10^6.5^ ELD_50_. The vaccination and challenge experiment with the chickens was conducted at the National Veterinary Institute, Ethiopia.

### 2.9. Statistical Analyses

Mean antibody or HI titers as well as mean values of optical density were calculated from the serum analysis of the individual animals in each experimental group and trial as described. If data followed a normal distribution, an analysis of variance (ANOVA) was employed for each time point under study and mean values were compared using the Tukey’s Multiple Comparison test. Otherwise, mean values were compared with the nonparametrical Kruskal–Wallis test and the level of significance and multiple comparisons were performed using the Dunn’s posttest. All tests were conducted using the statistical software GraphPad Prism v6.0.

## 3. Results

### 3.1. Antigenically Matched F and HN Antigens for Vaccine Design

Four Newcastle disease virus field isolates collected during outbreaks which occurred between the years 2013 and 2018 in different regions of Ethiopia were subjected to sequence analysis. One fragment of 749 bp corresponding to a variable region in the F protein sequence was obtained from each isolate. The sequences have been deposited at Genbank and were used in this study for sequence alignment and phylogenetic analysis. Construction of the phylogenetic tree involved these four Ethiopian isolates as well as strains presently being used in the manufacture of live attenuated vaccines at the National Veterinary Institute (NVI), Ethiopia. Other NDV sequences available at Genbank, such as geographically related isolates and representative strains of the different genotypes, were also included. The new Ethiopian isolates were grouped and showed a high degree of identity with strains in genotype VI, class II, and with sequences isolated mostly within the last 10 years. Strains currently in use for the preparation of NDV live vaccines at NVI clustered under the distant genotype II, class II (Supplementary Material 2). These data allowed the molecular design and subsequent construction of adenoviral transfer vectors for recombinant vaccine production bearing sequences of genotype-matched F and HN antigens.

### 3.2. Construction of Adenoviral Vectors Carrying the Foreign F and HN Gene Sequences from NDV

**Figure 1:**
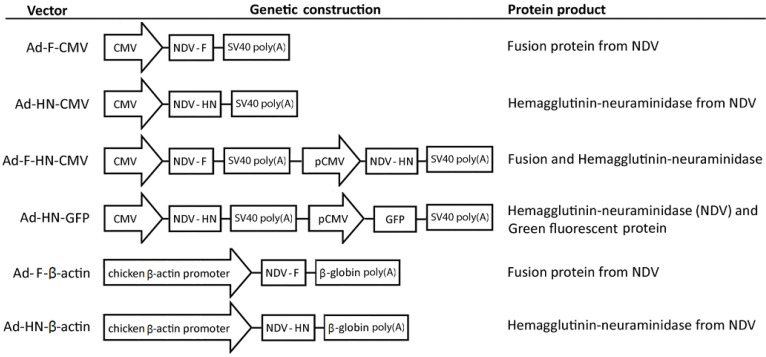
Nonreplicating chicken codon-optimized recombinant adenovirus constructions as vaccine candidates against Newcastle Disease virus (NDV): The schematic representation of the adenoviral constructs shows the regulatory regions for the control of expression and the fusion and hemagglutinin-neuraminidase proteins of NDV. The expression of the foreign antigens is driven either by a fragment of the human Cytomegalovirus (CMV) enhancer/promoter or by the avian β-actin promoter, as indicated. The adenoviral vectors have been designed to carry one or two (head-to-tail oriented) expression units for the individual expression or the co-expression of the NDV antigens. One of the constructs carries both the hemagglutinin-neuraminidase protein and the green fluorescent protein for monitoring and quantification during the process development steps implemented at different scales.

The adenoviral transfer vectors constructed for subsequent virus assembly and rescue were validated by restriction analysis, nucleotide sequencing, and finally by expression in vitro of the encoded antigens, corroborated by immunodot and Western blot (Supplementary Material 3). In the final adenoviral vectors generated, the expression of the F and HN antigens was driven by the human CMV promoter or the chicken β-actin promoter either as individual antigens or co-expressed in separated expression units. One additional viral construct expressing both the HN antigen and GFP allowed us to evaluate by flow cytometry the various experimental manufacturing steps and culture parameters of the adenovirus-vectored vaccines in order to establish a range of operating conditions. The regulatory elements and NDV genes in the genetic constructions are represented in Figure 1.

The recombinant adenoviruses were obtained as described in the Materials and Methods section and were rescued after seven days via transfection in HEK293A. Cell lysis was performed for Ad recovery and its use in further amplification steps. The identity of the adenoviral vectors was confirmed by PCR with amplification of the band sizes expected and detection of the recombinant products in HEK293SF-infected cells. For storage and further immunological evaluations, the viruses were purified in a step CsCl ultracentrifugation gradient and the concentration was calculated by the HPLC profile using UV detection. Cytopathic effect was visualized after infection of HEK293A cells in 96-well plates. The functional (infectious) titer for every stock was determined, resulting in values around 1–2 × 10^8^ TCID_50_/mL (approximately 10^10^ VP/mL).

### 3.3. Adenovirus Production and Operating Culture Conditions in Shake Flasks

**Figure 2:**
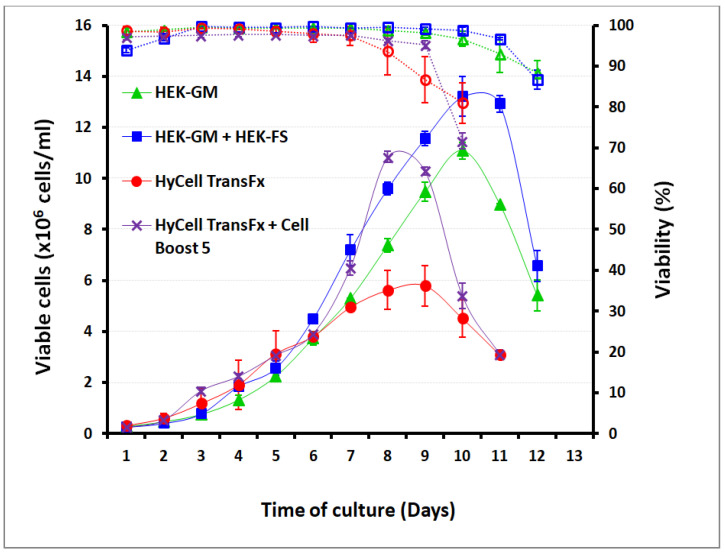
Effect of two different culture media and feeding supplements on HEK293SF cell density and viability (in percent) in shake flask experiments: HyCell TransFx-H media (Hyclone Laboratories Inc) and HEK-GM (Xell AG, Bielefeld, Germany) were used in the assay for comparative purposes, including the use of feeding supplements following essentially each manufacturer’s recommendations. Supplements consisted of a bolus addition. The feeding supplement HEK-FS (Xell AG, Bielefeld, Germany) was added starting from day 2 of the culture at 3% (v/v), then increasing to 4, then 5% (v/v), and finally 10% until the end of the culture at day 12. The HyCell TransFx-H medium was supplemented with CellBoost 5 (GE Healthcare, Chicago, IL, USA, USA) every two days by adding the feeding bolus at 5% (v/v). The higher cell densities were achieved with the HEK-GM basal medium and with the HEK-GM + HEK-FS combination, in which 1 × 10^7^ cells/mL and 1.3 × 10^7^ cells/mL were reached, respectively, after 9 days in culture. High cell densities (around 1 × 10^7^ cells/mL) were also reached by day 8 with the combination of HyCell TransFx-H and its feeding supplement. The complete assessment was conducted in duplicate, and each point corresponds to the mean ± standard deviation (bars in both senses are shown in the figure).

For experimental viral production in shake flasks, the effect of culture media and feeding supplements on cell density and viability of HEK293SF cells was first analyzed. Two media, HyClone HyCell TransFx-H and Xell AG HEK-GM, were assayed for this purpose. Cells in both media exhibited an exponential growth profile. Cells cultured in HEK-GM reached densities over 1 × 10^7^ cells/mL after 9 days in culture, while HyCell TransFx-H supported a maximum cell density of up to 5.7 × 10^6^ cells/mL after 8 days of culture. The highest cell densities, over 1.3 × 10^7^ cells/mL, were reached with the combination HEK-GM (basal medium) + HEK-FS (feeding supplementing), slightly outperforming the combination of HyCell TransFx-H plus Cell Boost™ 5 as feed. HyCell TransFx-H supported cell growth over 1 × 10^7^ cells/mL by day 10 of the culture when the feeding supplement was administered (Figure 2).

Infection experiments were conducted with the GFP-expressing adenoviral vector to enable rapid monitoring of the viral production. HEK293SF cells were cultured in batch in different shake flasks until reaching densities of 1, 2, 4, or 6 × 10^6^ cells/mL, at which time adenoviral infection was initiated. In this set of experiments, cells cultured in HyCell TransFx-H did not reach the cell density of 6 × 10^6^ cells/mL; therefore, no sample could be assayed at that point. Using identical cultures in parallel, feeding was provided at the time of infection at the respective cell concentrations. This experiment assessed the potential benefits of using a fed-batch strategy for culture intensification and for possible alleviation of limitations associated with a decrease in virus per cell production yield as the cell density set for infection increased.

**Figure 3:**
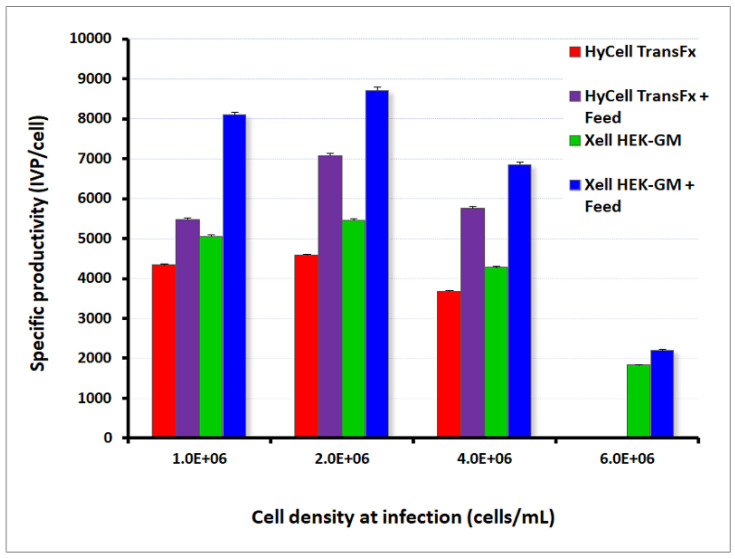
Adenovirus production in shake flasks experiments and evaluation of cell culture parameters and infection conditions for increased virus per cell production yields: Infection experiments were conducted with the recombinant Ad-HN-GFP-CMV adenoviral vector in order to monitor parameters in both phases of the culture. Two different culture media were evaluated: HyCell TransFx-H and Xell AG HEK-GM. In this approach, cells were cultured in batch until reaching densities of 1, 2, 4, and 6 × 10^6^/mL (this last value is only for HEK-GM), and viral infection was initiated at each of these points. The effect of cell density on the specific cell yield was evident when a decrease in specific production was observed at higher cell densities, indicating a metabolic limitation. A feed supplement was added at each cell concentration at the time of infection, using paralleled cultures and following the regimens explained in Materials and Methods. It was demonstrated at every cell density analyzed that feeding significantly increased the specific production yield (IVP/cell) of the cultures compared to the non-supplemented cultures. The highest values of cell specific yields (calculated from fluorescence values detected by flow cytometry) were reached in both media by infection at 2M cells/mL. The combination HEK-GM + HEK FS resulted in the highest specific production and was used either in shake flasks or for the scale-up to bioreactors. The experiment was conducted in duplicate and deviations between replicates are indicated in the figure by standard deviations bars in the positive sense.

It was first demonstrated at every cell density analyzed, with the two culture media evaluated, that feeding contributed to increasing the specific production yield (IVP/cell) of the cultures compared to the non-supplemented cultures. This increase was similar in both media (increase of around 1.6-fold) and was detected at all the cell densities analyzed in the case of the Xell AG HEK-GM medium. An indication of metabolic limitation was observed when comparing specific cell yields in cultures infected at 1 × 10^6^ cells/mL versus the ones infected at higher cell density. The cell-specific yield decreased with an increase of the cell density at infection, which was partially alleviated with the feeding strategy. The highest specific cell production values were obtained in both media by infecting at the cell density of 2 × 10^6^ cells/mL with the addition of the feed supplement. The combination of HEK-GM basal medium plus HEK-FS feeding resulted in a superior outcome as compared to the use of HyCell TransFx-H and its supplement (mean values of 8720 vs. 7080 IVP/cell, respectively) (Figure 3).

Virus titers in the cell culture lysate supernatants (volumetric yield) were also superior when a supplemented feeding was used, as compared to non-supplemented cultures. Maximum values were also reached for the HEK-GM medium in cultures infected at a cell density of 2 × 10^6^ cells/mL (5.57 × 10^8^ IVP/mL without feed versus 9.52 × 10^8^ IVP/mL with feed). With these experiments, specific operating culture conditions were identified and used for the subsequent productions in controlled bioreactor runs, including the selection of the HEK-GM medium for virus production.

### 3.4. Scale-Up for Adenovirus Production In 1 and 3 L Bioreactors

**Figure 4:**
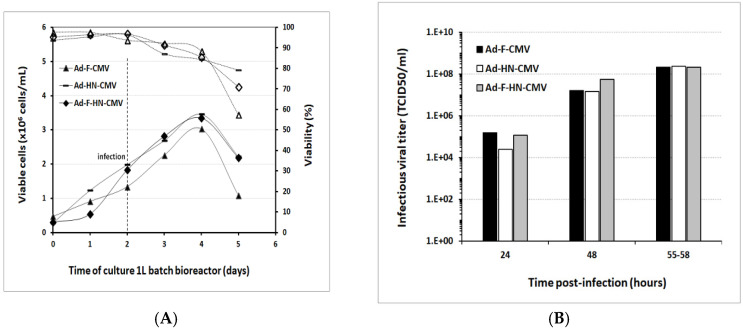
Adenoviral vector production in 1 L controlled bioreactors operated in batch mode: (**A**) Time course of viable cell growth and cell viability during the production of the three recombinant adenoviral vectors encoding the F, HN, and F-HN antigens from NDV under the regulation of the CMV enhancer/promoter. The culture medium was Xell AG HEK-GM and the operational parameters were described in the Materials and Methods section. The time of infection was set at a cell density of 2 × 10^6^ cells/mL as indicated in the figure, and the cultures were harvested when cell viability reached approximately 60–70%. The cell density data are shown by solid symbols and solid lines, and the percentage of cell viability is shown by empty symbols and dotted lines. The infections were conducted with the different adenoviral vectors; ∆, Ad-F-CMV; —, Ad-HN-CMV; ◊, Ad-F-HN-CMV. (**B**) Production of infectious viral particles per mL in the cell culture lysate supernatants analyzed at different time points using culture samples taken during the run. Calculations of TCID_50_/mL values were performed as described. They were in the range of 1.3 to 2.2 × 10^8^ TCID_50_/mL.

In order to evaluate the scalability of the results obtained from the 40 mL shake flasks, the production of the recombinant adenoviruses was undertaken in serum-free suspension HEK293 cells, using Xell AG HEK-GM medium in 1 L and 3 L bioreactors. First, three 1 L batch bioreactor runs were set up for the production of the Ad-F-CMV, Ad-HN-CMV, and Ad-F-HN-CMV vectors to generate material for immunologic evaluations in the mice model. The HEK293SF cells were seeded at approximately 0.35 × 10^6^ cells/mL in a 750 mL bioreactor working volume and grown until rapidly reaching a cell density of approximately 2 × 10^6^ cells/mL. At this stage, the cells were infected with each virus stock at a MOI of 1. Cell concentration slightly increased after virus infection until reaching a maximum in the range of 3.4–4 × 10^6^ cells/mL and rapidly started to decrease along with a decline in cell viability (Figure 4). Cells were harvested when the cell viability reached 60–70%, which occurred at around 55 hpi. The cultures were sampled daily, and infectious particle determination was performed. Titers at the time of harvest in the cell culture supernatants ranged from 1.80 to 2.17 × 10^8^ TCID_50_/mL, with corresponding values of total viral particles from 2.6 to 4.8 × 10^10^ VP/mL (Table 1). The CsCl-purified preparations in the final formulation buffer resulted in total viral particles in the range of 10^10^–10^11^ VP/mL and 1.8–4.0 × 10^9^ TCID_50_/mL. The ratio of infectious viral particles to total viral particles in the cell culture lysate supernatants was below 1%. This value increased from 3- to 6-fold after concentration and purification into the final formulation buffer in the vaccine stocks. These results demonstrated the potential scalability of virus production in controlled bioreactors, with growth and production profiles comparable to those observed in shake culture flasks.

**Figure 5:**
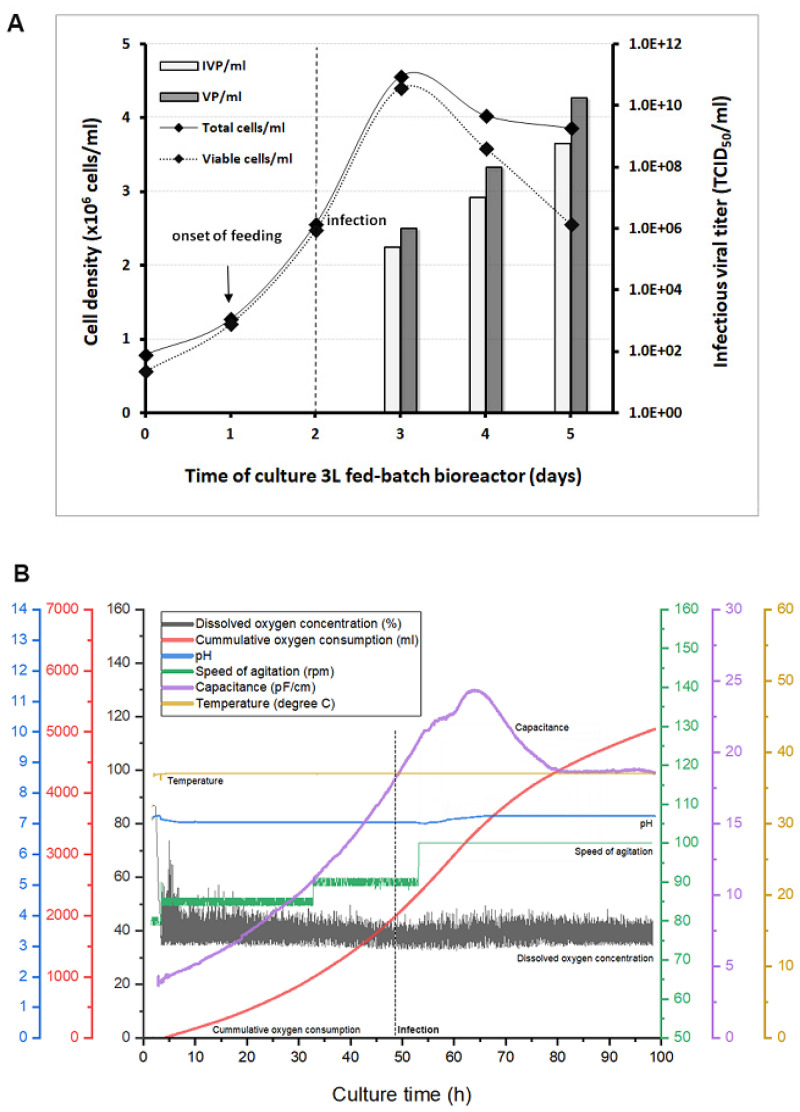
Adenoviral vector production in a 3 L controlled bioreactor operated in fed-batch mode for the production of one adenoviral vector as vaccine candidate: (**A**) Time course of the viable and total cell growth in Xell AG HEK-GM medium in which the feed supplement was initiated at a cell concentration of 1 × 10^6^ cells/mL as indicated in the figure. The infection was set at a cell density of 2 × 10^6^ cells/mL, and the final cell density during the virus production phase reached 4.56 × 10^6^ cells/mL. The cultures were strictly monitored and harvested when the cell viability reached approximately 60–70%. At harvest, the infectious viral particles of the Ad-F-HN-CMV produced reached 5.8 × 10^8^ TCID_50_/mL in the cell culture lysate supernatants. In addition, the curve of the capacitance values (**B**) measured in-line shows the evident changes registered after infection with the adenoviral vector as the cells undergo the productive phase. The profile of various bioreactor sensors and process parameters are also shown (speed of agitation, pH, dissolved oxygen concentration, cumulative oxygen, and temperature). The onset of feeding supplementation is indicated with an arrow in panel A. The dotted line in both panels indicates the time point of infection and the shift in the culture phase.

Thereafter, fed-batch cultures in 1 and 3 L bioreactors were implemented for the preparation of larger amounts of highly purified working lots for vaccination and demonstration of protective efficacy in the target species. The process scale-up and validation of fed-batch bioreactors were based on repeated productions runs that were monitored and operated with the purpose of improving production yields compared to bioreactors run in batch mode. The operating conditions (pH, temperature, dissolved oxygen, and feeding strategy) were also extensively documented for the establishment of standard operating procedures. As an example, the cell growth profile and the kinetics of the recombinant adenovirus production, measured as total and infectious viral particles, are provided in Figure 5A for one 3 L culture implemented. Viral production was detected at 24 hpi, and viral titers kept rising until the cell density reached 4.5 × 10^6^ cells/mL. In the various runs performed, cell density and viability started to decrease within the first 24 h period after infection while infectious titers were measured consistently at around 5–6 × 10^8^ TCID_50_/mL (1.08 to 2.24 × 10^10^ VP/mL) at harvest after 48 hpi. The bioreactors were harvested when the cell viability reached 60–70%. In the final vaccine preparations, total viral particles were in the range of 1.1 to 7.01 × 10^11^ VP/mL and infectious viral particles were in the range of 1.03 to 6.3 × 10^10^ TCID_50_/mL. The IVP-to-VP ratio in cell culture lysate supernatants was improved in fed-batch bioreactors, with values of around 3–5%. After purification and concentration steps, the IVP/VP ratios encountered ranged from 12 to 22%. The repeatability of the process is summarized in Table 2 and provides solid evidence of its robustness, thus supporting the potential scale-up and industrialization of the process. When the 3 L bioreactor was run, in-line monitoring of the capacitance probe signal was also included. This is an indicator of the total biovolume of the culture and a marker of cell physiological changes during the process. The variations in capacitance and conductivity were indicators of the total biomass during the growth and indicated the cells shift to the viral production phase (Figure 5B).

### 3.5. Immunogenicity and Protective Efficacy Assessments of the NDV Adenovirus Vaccine Variants

**Figure 6:**
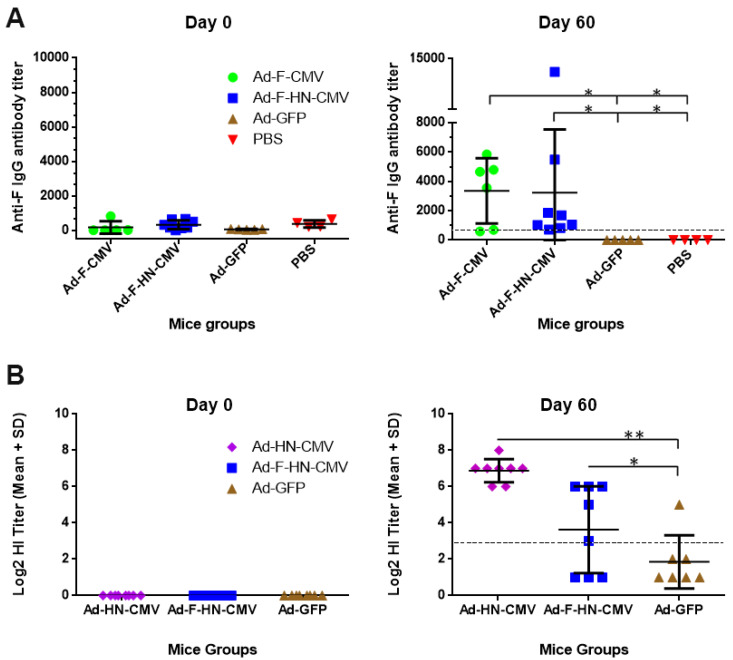
Analysis of the humoral immune response generated in mice against NDV after vaccination with the recombinant adenoviral vectors Ad-F-CMV, Ad-HN-CMV, Ad-F-HN-CMV, and Ad-GFP-CMV (negative control): Enzyme-Linked Immunosorbent Assays (ELISA) and Hemagglutination Inhibition Assay (HIA) assays were conducted for the detection of anti-F IgG antibodies and antibodies with HI activity, respectively, in the serum of vaccinated mice. Mice were immunized via i.m. injections at days 0 and 21 with doses of 10^7^ TCID_50_. (**A**) The mean ± standard deviations of anti-F IgG antibody titers measured at days 0 and 60 of the experiment. Specific titers elicited by the Ad-F-CMV and the Ad-F-HN-CMV vectors increased over the indicated cutoff (>993) for existence of protective immunity (dotted line). Both groups of animals, injected with the Ad-F-CMV or Ad-F-HN-CMV viruses, showed statistically significant differences compared to the negative controls. (**B**) The generation of antibodies with hemagglutination inhibition capacity in vitro is shown. Mean titers were calculated from values of antibodies with HI activity detected in serum of animals using the logarithm base 2 of the titer. In animals vaccinated with the Ad-HN-CMV and Ad-F-HN-CMV, this response was statistically superior to measurements in the negative control group. According to the routine practices at the NVI, different degrees of protection against NDV are achieved in chickens when HI titers over Log_2_(3) (dotted line). Statistically significant differences (*p* < 0.05) are represented with one asterisk in the figure and two for *p* < 0.01.

The first lots of adenoviral vectors produced in 1 L bioreactors in batch mode were purified and formulated. They were utilized in one vaccination experiment conducted in mice to evaluate the immunogenicity of the constructs that express the NDV antigens under the control of the CMV promoter, which is preferentially used for the expression of proteins in mammalian cells. Groups of mice were immunized twice intramuscularly with a dose of 10^7^ TCID_50_ of each vector (Ad-F-CMV, Ad-HN-CMV, Ad-F-HN-CMV, and Ad-GFP), and serum samples were collected and assayed for specific antibodies until day 60 after the primary immunization. ELISA and HIA were performed to detect specific anti-F IgG or antibodies with hemagglutination inhibition activity in mice sera. Mice vaccinated with the Ad-F-CMV and the Ad-F-HN-CMV vectors developed mean anti-F antibody titers that rose over 1/3000, with most of the animals over the titer cutoff of 1/993. This value correlates with the onset of protective efficacy, according to the manufacturers of the assay (Figure 6A). Statistically significant differences were encountered between the vaccinated animals and the negative controls groups, consisting of animals injected with Ad-GFP or PBS. Although no statistically significant differences were detected between the groups immunized with the F and F-HN encoding viruses, the F protein expressed as a single antigen seemed to induce a superior and more homogeneous response of specific antibodies.

HI assays showed the detection of antibodies with in vitro hemagglutination inhibition activity against NDV. The HI activity was maintained until day 60 of the experiment in mice vaccinated with the Ad-HN-CMV and Ad-F-HN-CMV vectors. Assessments at days 0 and 60 of the experiment are shown in Figure 6B. HI titers with activity over log_2_(5) as well as statistically significant differences (*p* < 0.01) with respect to the negative control were detected. A suggested cutoff value (according to NVI practice criteria) for the presence of specific protective immunity in chickens corresponds to HI titers ≥ log_2_(3). By the end of the experiment, no adverse effects due to vaccination could be recorded in the groups of mice immunized.

**Figure 7:**
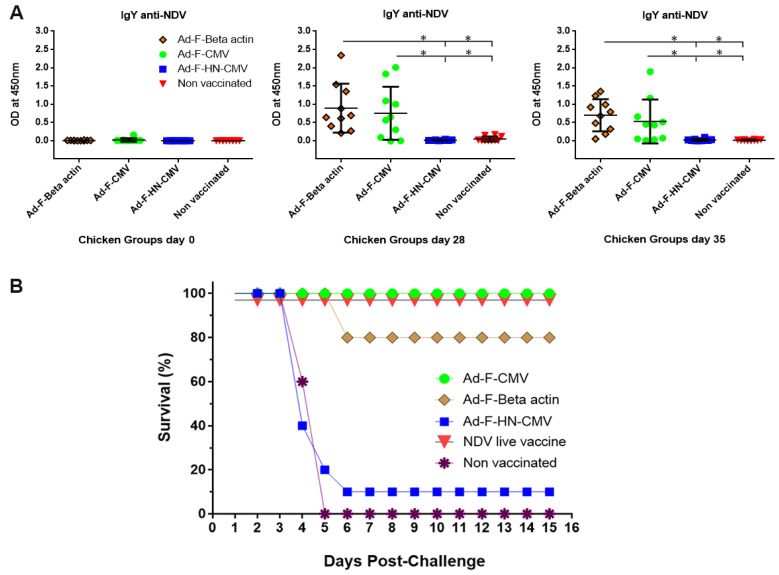
Analysis of the humoral immune response generated in chickens against NDV-encoded antigens and assessment of the protective efficacy: The level of anti-NDV antibodies after immunization with the recombinant adenoviral vectors Ad-F-β-actin, Ad-F-CMV, and Ad-F-HN-CMV were evaluated by ELISA (**A**) for the detection of IgY-specific antibodies in the serum of chickens vaccinated via i.m. with two doses of 10^10^TCID_50_. Mean optical values were calculated from serum of individual animals. The figure shows the means ± standard deviations of optical density values representing the S/P ratio calculated as previously described. Specific antibodies were detected until the last determination, prior to the lethal challenge with NDV and were found to be over the S/P cutoff value of 0.3, one indicative of protective efficacy. Specific antibodies were not detected in the negative control groups. Statistically significant differences (*p* < 0.05) are represented in the figure with an asterisk. (**B**) Eight weeks after the primary immunization, all chickens were intramuscularly challenged with 0.5 × 10^6.5^ ELD_50_ of the NDV isolate Debre/zeit/2018 (MN909678). A positive control group vaccinated with NDV live vaccine according to the manufacturer’s instructions as well as one group of non-vaccinated animals were included in the challenge. All chickens were monitored for 15 days to record the appearance of clinical signs of the disease and the total number of deaths. After this period, the percent survival was 100% for the groups vaccinated with the Ad-F-CMV and the NDV live vaccine, 80% for the group of chickens receiving the Ad-F-βactin vector, and 10% in the group vaccinated with the Ad-F-HN-CMV. One hundred percent of mortality and the typical signs of the disease were recorded in the group of non-immunized animals.

One experiment was then conducted in groups of chickens seronegative to NDV in order to evaluate the capacity of the adenoviral vectors to elicit a specific immune response in the target animal species and the degree of protection conferred. Vaccination lots prepared in fed-batch 1 and 3 L bioreactors were used for this purpose. The adenoviral vectors were produced, CsCl-purified, concentrated, formulated, and stocked at infectious titers in the order of 5 × 10^10^–10^11^ TCID_50_/mL. Ten chickens per group were vaccinated with the adenoviral vectors Ad-F-βactin, Ad-F-CMV, Ad-F-HN, and the NDV thermostable vaccine. One last group was left unvaccinated. Two doses of 10^10^ TCID_50_ adenovirus vaccine were intramuscularly administered, and serum samples were taken periodically to assess the generation of specific humoral immune responses to NDV. The vaccines provided were able to generate anti-F specific IgY antibodies 14 days after the primary immunization as detected by ELISA. The optical density (OD) values with a signal to positive (S/P) ratio > 0.3 were considered as an indicator of immunity against NDV, suggesting the onset of protection. Vaccination elicited this level of response in all the animals immunized with the vectors Ad-F-β-actin and Ad-F-CMV (Figure 7A). This was significantly different when compared to unvaccinated animals. No significant levels of specific antibodies to NDV antigens could be demonstrated in chickens immunized with the Ad-F-HN vector. An intramuscular NDV lethal challenge was finally administered at day 56 of the experiment. Chickens were daily monitored for 15 days for changes in behavior, for appearance of clinical signs of the disease, and for adverse effects attributable to vaccination, including observation at the sites of injection. The mortality was recorded shortly after the challenge starting from day three after exposure to NDV and continuing until day 6 in those groups where mortality occurred. After the sixth day post-challenge, no additional deaths were recorded. The groups of chickens immunized with the Ad-F-CMV and Ad-F-β-actin vectors showed survival rates of 100 and 80%, respectively. All the animals vaccinated with the NDV live vaccine survived the challenge as well (Figure 7B). Low survival was observed in animals immunized with the Ad-F-HN vector. In the group of unvaccinated chickens, all animals died showing the characteristic clinical signs of the disease such as depression, loss of appetite, recumbent behaviour, paralysis, and death. Postmortem examination of these animals showed the presence of proventricular haemorrhages. Vaccinated chickens that did not survive the viral challenge showed the characteristic progression of ND as well. Immunized chickens which survived the challenge showed specific anti-NDV IgY antibodies but undetectable hemagglutination inhibition activity in vitro. However, they showed no clinical signs of the disease during the whole observation period, and no specific lesions attributed to NDV infection were observed after euthanasia and necropsy examinations.

## 4. Discussion

Newcastle Disease (ND) is a permanent threat that negatively impacts poultry producers worldwide despite the use of live-attenuated and inactivated vaccines [32]. The disease can affect over 236 species of birds [33] and remains one of the most critical diseases for poultry species, close to the impact of avian influenza [34]. In sub-Saharan Africa, ND outbreaks with high mortality have massively affected the potential of villagers to raise chickens [10,35]. Effective ND control could potentially improve rural farmers’ capabilities to maintain and develop chickens production, playing an important role in food security and in reduction of poverty [36]. Although the goal of vaccination is sterilizing immunity, this has not been totally achieved since the amount of NDV shed into the environment remains a persistent problem worldwide [37]. Even though all strains of NDV belong to one serotype, there are phylogenetic differences when comparing the genomes from different isolates [17]. Various studies have demonstrated that vaccines formulated with strains more similar to the challenge virus (actual circulating strains) can be more effective in decreasing the amount of challenge virus shed from vaccinated birds [38,39,40,41]. In this work, the study of locally circulating stains enabled the design of genotype-matched vaccines in correspondence with NDV genotype VI present in the geographical region considered. It has been previously demonstrated that the fusion protein is the major contributor to protective immunity of genotype-matched vaccines in NDV vaccination strategies [13,42]. The degree of cross protection against multiple strains after vaccination with antigens from currently circulating viruses (such as of class II, genotypes V, VI, VII, and XII–XVIII) should be also superior than cross protection generated by historical and phylogenetically distant strains isolated prior to 1960.

An analysis of the ND outbreaks at a global scale suggests that new approaches for NDV vaccine generation are needed. Recombinant and cell culture technologies appear as the most promising strategies for the rapid development of new vaccine candidates to rapidly respond to the reality of different circulating genotypes. More importantly, the introduction and development of such technologies and production platforms in the East African region is also supported by the need for the rapid production of vaccines in preparation for response to emerging pathogen outbreaks, in particular, pathogens with high levels of confinement required for the development of traditional vaccines. Adenovirus vectors are known to induce strong humoral, mucosal, and cellular immune responses to the encoded antigens. Based on data generated in numerous clinical protocols, adenovirus vectors are considered very effective vectored vaccines [43,44] and their mass production has been previously documented in an important number of studies [20,45,46,47], seeking significant improvements to reduce the cost of goods and to achieve economical viability. In this study, the production process supported cell growth of HEK293 cells to a density over 4.5 × 10^6^ cells/mL during the virus production phase, in which high levels of volumetric and specific cell production yields were achieved. It was also observed in shake flasks experiments cultured in batch that Ad infection at increasing cell densities resulted in specific production values that decreased with the increment in cell density, which is characteristically referred to as the ‘‘cell density effect’’ [28] and is attributed to nutrient depletion and accumulation of inhibitory metabolites. However, it was demonstrated here that higher specific cell yields could be obtained with the addition of feed supplements, which will contribute to achieving high-yielding fed-batch productions at larger scale. Infecting cultures at higher cell densities while maintaining the specific productivity is a major desirable goal in viral vector production. The fed-batch culture operating mode combines both operational simplicity and higher product yields compared to batch and perfusion processes [48,49]. Previous studies on recombinant Ad5s have demonstrated the positive effects of medium exchange or medium addition with important improvements in both VP/mL and IVP/mL concentrations [46].

Although vaccination is an effective control strategy against ND, conventional vaccination usually faces challenges related to the cold-chain and vaccine administration methods [2,35]. In this sense, an adenoviral vector-based vaccine could also benefit from being prepared in a lyophilized form, a well-established technology for adenovirus products that would allow a more convenient distribution with reconstitution at the time of administration [50]. Regarding its potential for commercial use, some successful adenovirus vector vaccines have recently demonstrated their value and applicability in the field, such as the live adenovirus vector anti-rabies vaccine (Ontario Rabies Vaccine Bait; ONRAB) [51] and the extensively studied and licensed-for-marketing adenovirus type-5 vector-based Ebola vaccine (Ad5-EBOV) [52].

The main objective in this work has been to streamline and demonstrate a successful production process and scale-up for manufacturing of an adenovirus-vectored ND vaccine, supported by a large set of reproducible data. For a robust manufacturing process, including the technology transfer to the region, a set of bioreactor monitoring tools, control strategies, and protocols for viral product quality control were developed and have been reviewed here. The critical process parameters were identified and assessed during the process validation and have been documented in a set of standard operating procedures. Currently, ongoing studies are also showing promising results on scalable extraction steps from the bioreactor, such as cell disintegration by chemical agents and filtration to replace the centrifugation and freeze-thaw lysis steps, followed by tangential flow filtration in order to reduce labor costs associated with the manufacturing process. Overall, this will translate to cost-effectiveness, which is a key success factor for a veterinary vaccine [47,53].

Conferring a solid protective efficacy is another primary requirement for a successful vaccine. Preliminary challenge experiments conducted in groups of chickens at NVI, Ethiopia, suggested a superior role in protection for adenoviral constructions bearing the F protein over the HN protein. Results were found later with the Ad-F-CMV vaccination protocol, where 100% protection was achieved without any clinical signs of the disease. It is interesting to note that no hemagglutination inhibition titers were detected in the chickens fully protected by vaccination. This protection could have been mediated by antibodies produced against the F protein that neutralize the virus by binding and preventing attachment to the host cells [43,54,55]. This reinforces a key role of the F antigen in its ability to induce a sufficiently potent response against NDV. Future animal experiments will also aim for mucosal delivery alternatives, dose-protection studies, and specific assessments on the onset and duration of protection, including a more detailed characterization of the humoral and cell-mediated immune response generated. These experiments may also aid in providing a rational explanation to the differential degree of protection generated by the two viral vectors encoding the F antigen. Importantly, additional studies taking into consideration the societal and geographical environments will provide the fundamental bases to establish the regulatory requirements and to support the deployment of an effective and safe recombinant adenovirus-based poultry vaccine in sub-Saharan Africa.

## Figures and Tables

**Table 1:** Summary of key process data and recombinant adenovirus productivity in the 1 L bioreactors operated under batch culture mode.

Culture Mode in Bioreactor	Batch 1 L	Batch 1 L	Batch 1 L
Adenovirus produced	Ad-F-CMV	Ad-HN-CMV	Ad-F-HN-CMV
Peak of cell density in the production phase	3.42 × 10^6^ cells/mL	4.01 × 10^6^ cells/mL	3.93 × 10^6^ cells/mL
Total cell density at harvest	1.88 × 10^6^ cells/mL	2.76 × 10^6^ cells/mL	3.08 × 10^6^ cells/mL
Viability at harvest	57.4%	79.1%	71.0%
Total viral particle concentration in cell culture lysate supernatants	4.80 × 10^10^ VP/mL	3.45 × 10^10^ VP/mL	2.64 × 10^10^ VP/mL
Infectious viral particle concentration in cell culture lysate supernatants	2.17 × 10^8^ IVP/mL	1.29 × 10^8^ IVP/mL	1.80 × 10^8^ IVP/mL
IVP/VP ratio in cell culture lysate supernatants	~0.4%	~0.4%	~0.7%
Total viral particle concentration in CsCl purified samples	1.08 × 10^11^ VP/mL	1.02 × 10^11^ VP/mL	1.0 × 10^11^ VP/mL
Infectious viral particle concentration in CsCl purified samples	3.21 × 10^9^ IVP/mL	4.06 × 10^9^ IVP/mL	1.86 × 10^9^ IVP/mL
IVP/VP in CsCl purified, concentrated vaccine stocks	~3%	~4%	~2%

**Table 2:** Summary of key process data and recombinant adenovirus productivity in the 1 L and 3 L bioreactors operated under fed-batch culture mode.

Culture Mode in Bioreactor	Fed-Batch 1 L	Fed-Batch 1 L	Fed-Batch 1 L	Fed-Batch 3 L	
Adenovirus produced	Ad-F-CMV (1)	Ad-F-CMV (2)	Ad-F-βactin	Ad-F-HN-CMV	Ad-F-βactin
Peak of cell density in the production phase	4.28 × 10^6^ cells/mL	4.35 × 10^6^ cells/mL	3.87 × 10^6^ cells/mL	4.56 × 10^6^ cells/mL	3.81 × 10^6^ cells/mL
Total cell density at harvest	4.13 × 10^6^ cells/mL	4.18 × 10^6^ cells/mL	3.08 × 10^6^ cells/mL	3.86 × 10^6^ cells/mL	1.86 × 10^6^ cells/mL
Viability at harvest	78.4%	80.1%	60.7%	66.5%	76.2%
Total viral particle concentration in cell culture lysate supernatants	1.08 × 10^10^ VP/mL	2.24 × 10^10^ VP/mL	1.19 × 10^10^ VP/mL	1.78 × 10^10^ VP/mL	1.58 × 10^10^ VP/mL
Infectious viral particle concentration in cell culture lysate supernatants	5.01 × 10^8^ IVP/mL	5.64 × 10^8^ IVP/mL	3.2 × 10^8^ IVP/mL	5.80 × 10^8^ IVP/mL	5.03 × 10^8^ IVP/mL
IVP/VP ratio in cell culture lysate supernatants	~5%	~2.5%	~2.7%	~3.3%	~3.2%
Total viral particle concentration in CsCl purified samples	7.01 × 10^10^ VP/mL	5.23 × 10^10^ VP/mL	1.36 × 10^11^ VP/mL	5.35 × 10^11^ VP/mL	1.11 × 10^11^ VP/mL
Infectious viral particle concentration in CsCl purified samples	1.03 × 10^10^ IVP/mL	1.06 × 10^10^ IVP/mL	3.2 × 10^10^ IVP/mL	6.30 × 10^10^ IVP/mL	2.46 × 10^10^ IVP/mL
IVP/VP ratio in CsCl purified, concentrated vaccine stocks	~15%	~20%	~22%	~12%	~22%

## Supplementary Materials

The following are available online at https://www.mdpi.com/2076-393X/8/2/338/s1.

## Abbreviations

Ad: Adenovirus, CMV: Cytomegalovirus, DF1: chicken fibroblast cell line, DO: dissolved oxygen, ELISA: Enzyme-linked immunosorbent assay, GFP: Green fluorescent protein, HEK: Human embryonic kidney, HIA: Hemagglutination inhibition assay, IVP: Infectious viral particles, MOI: Multiplicity of infection, NDV: Newcastle Disease virus, OD: optical density, PEI: Polyethylenimine, PCR: polymerase chain reaction, TCID_50_ Assay: Median Tissue Culture Infectious Dose, VP: Viral particles.
