# Supplementary material for: Establishing a Robust Manufacturing Platform for Recombinant Veterinary Vaccines: An Adenovirus-Vector Vaccine to Control Newcastle Disease Virus Infections of Poultry in Sub-Saharan Africa
Source: Vaccines (Basel). 2020 Jun 26;8(2):338. doi: 10.3390/vaccines8020338 (PMC7350225; doi:10.3390/vaccines8020338)

vaccines

MDPI

Supplementary Material 1. Nucleotide sequences used for the construction of the vaccine vectors and oligonucleotide primers for characterization of the adenoviruses obtained. The Debre zeit sequence was used as reference in conjunction with the isolate APMV-1/Ethiopia/13VIR3936-27/2012 [KJ958914] for completion of the Fusion (1662 bp) and Hemagglutinin-neuraminidase (1737 bp) gene sequences. PCR reactions were also performed for confirmation of the identity of the adenoviral vectors after infection and extraction of viral DNA from the culture supernatants of HEK293SF cells. The oligonucleotide primers are summarized below.

F gen

ATGGGCTCCAGATCTTCTACCAAAATCCCAGCACCTGTGACGCTGACTGCCCAAATTATGTTGATATTG
AGCTGTATCTGTCCGACAAACCCTCTTGACGGCAGGTCTCTTGCAGCTGCAGGAATTGTGGTAACAGG
AGACAAAGCAGTTAATATATACACCTCATCTCAGACGGGATCAATCATAGTCAAGTTGCTCCCAAACA
TGCCCAAAGATAAAGAGGCATGTGCAAAAGCCCCATTAGAAGCATACAACAGAACACTGACCGCTTT
ACTCACCCCTCTCGGTGACTCTATCCGCAGGATACAGGGGTCCGTATCCAAGTCAGGAGGAGGAAGA
CAAGGACGCTTAGTAGGTGCCATTATTGGCAGTGTAGCTCTCGGGGTCGCAACATCAGCACAGATAAC
AGCAGCTGCAGCCTTAATACAAGCCAACCAGAATGCTGCTAACATCCTTCGGCTTAAGGAGAGCATTG
CTGCCACCAATGAAGCTGTGCATGAAGTCACTGACGGATTATCACAACTAGCTGTGGCAGTTGGTAAG
ATGCAGCAGTTTATTAATGACCAATTCAATAATACGGCGCGGGAACTGGACTGTATGAAAATTACCCA
ACAAGTCGGTGTAGAACTCAACCTATACCTAACTGAACTGACTACAGTGTTTGGGCCGCAAATCACCT
CCCCTGCCCTAACTCAGCTGACTATCCAGGCGCTTTATAATTTAGCTGGTGGCAATATGGATTACTTGC
TGACTAGGTTAGGCGTGGGGAACAATCAACTTAGCTCATTAATAAGTAGTGGCTTGATCACAGGCCAT
CCTATACTGTATGACTCACAAACTCAGCTCCTGGGCATACAAGTAAATTTACCCTCAGTCGGGAACCT
CAATAATATGCGTGCCACCTACTTGGAGACTATATCCGTAAGCACAACCAAGGGGTTTGCCTCAGCAC
TTGTCCCAAAGGTAGTGACGCAAGTCGGCTCCGTGATAGAAGAACTCGACACTTCACACTGTATAGAG
TCTGAACTGGATTTATATTGTACAAGAATAGTGACATTCCCCATGTCCCCTGGCATTTATTCCTGTCTGA
GCGGCAACACATCGGCTTGCATGTACTCAAAGACTGAAGGTGCACTCACTACGCCATATATGACCCTC
AAAGGCTCAGTTATTGCCAATTGCAAAATAACAACATGTAGGTGCGCAGACCCCCCAAGTATCATATC
GCAAAACTATGGAGAAGCTGTGTCTCTGATAGATAGACATTCATGCAATGTCCTGTCACTAGATGGGA
TAACTCTGAGGCTCAGTGGGGAGTTTGATGTGACTTATCAAAAGAATATCTCAATACTAGAATCTCAA
GTCATCGTGACCGGCAATCTTGATATATCAACCGAGCTAGGAAATGTCAACAATTCAATAAGCAGTGC
CCTAGGTAAGTTAGCGGAGAGTAACAGTAAACTAGACCAAGTCAATGTCAAACTGACCAGCACATCT
GCACTCATTACCTATATTGCTCTAACCACCATATCTCTTGTCTCCGGTATACTTAGCCTGGGTTTGGCGT
GCTACCTAATGTACAAACAAAAAGCACAACAAAAGACCTTAATATGGCTTGGGAATAATACCCTTGA
TCAGATGAGAGCTACTACAAGGACATGA

HN gen

ATGGACCGCGCAGTTAGCCAAGTTGCGCTAGAGAATGATGAAAGAGAGGCAAGGAATACATGGCGC
CTGGTATTCCGGATCGCAATCCTATTTTTAACGGTAGTGACTCTAGCCGTCTCTGCAGCTGCCCTTGCAT
ATAGTATGGAAGCCAGCACACCTAGCGATCTTGTAGGCATACCGACTGCGATCTCTAGAGCAGAGGA
AAAGATTGCATCTGCACTCGGTTCCAATCAAGATGTAGTAGATAGAATATATAAGCAGGTGGCCCTCG
AATCTCCACTGGCATTGCTAAACACCGAATCCACAATTATGAACGCAATAACATCTCTCTCTTATCAG
ATCAATGGGGCTGCAAATAGCAGCGGATGTGGAGCACCCATTCATGATCCAGATTATATTGGGGGAAT
AGGTAAAGAACTTATTGTAGATGATGATAGCGACGTCACATCATTCTATCCCTCCGCATTCCAAGAAC
ATCTGAACTTTATCCCGGCGCCTACTACAGGATCAAGCTGCACTCGGATACCCTCATTTGATATGAGTG
CTACCCACTACTGTTATACTCACAATGTGATATTATCTGGATGCAGAGATCACTCGCACTCACATCAAT
ATTTAGCACTTGGTGTGCTTCGGACATCTGCAACAGGGAGGGTATTCTTTTCCACTCTGCGTTCCATCA
ATCTGGATGACACCCAAAATCGGAAGTCTTGCAGTGTGAGTGCAACTCCCTTGGGTTGTGATATGCTAT
GTTCCAAAGTCACAGAGACTGAAGAAGAGGATTATAACTCAGCTGCCCCCACGTCGATGGTACACGG
GAGGTTAGGGTTCGACGGCCAATATCATGAGAAGGACCTAGACGTCACAACACTATTCGAGGACTGG
GTGGCAAACTACCCAGGAGTAGGGGGCGGGTCTTTTATTGACAATCGCGTATGGTTCCCAGTTTACGG
AGGGCTAAGACCCAATTCGCCCAGTGACACCGCACAAGAAGGGAAATATGTAATATACAAGCGATAT
AATGACACATGTCCAGACGAGCAAGATTATCAGATTCGAATGGCTAAGTCTTCATATAAGCCTGGGCG
GTTTGGTGGGAAACGCGTACAGCAAGCCATCTTATCTATCAAAGTGTCAACATCCTTGAGTGAGGACC
CAGTGCTGACTGTACCGCCCAACACAGTCACACTCATGGGGGCTGAAGGCAGAGTTCTCACAGTAGG
GACATCTCATTTCTTTTATCAACGAGGGTCATCGTACTTCTCCCCTGCCCTACTATACCCCATGATAGTC

AGTAACAAAACAGCCACTCTTCATAGTCCTTATATATTCAATGCTTTTACTCGACCAGGTAGTATCCCT
TGCCAGGCTTCAGCAAAATGCCCTAACTCATGTGTTACCGGAGTCTATACTAATCCATATCCCTTGGTC
TTCTATAGGAACCACACCTTGCGAGGGGTATTCGGGACGATGCTTGATGATGTACAAGCAAGACTCAA
CCCGGTATCTGCAGTATTTGACGGCATATCTCGCAGTCGCATAACCCGGGTGAGTTCAGGCAGCACCA
AGGCAGCATACACAACATCGACATGTTTTAAAGTTGTAAAGGCCAATAAAACCTATTGTCTCAGCATT
GCAGAAATATCCAATACCCTATTCGGGGAATTCAGAATCGTCCCTTTACTAGTTGAGATTCTCAAGGAT
GATGGGGTTAGAGAAGCCAGGTCTAGCCGGTAG

Oligonucleotide primers

F gene	Tm (℃)	Product Size (bp)
F- TTAGCTGGTGGCAATATGGA	59.1	221
R- AACCCCTTGGTTGTGCTTAC	59.0	
F- TGTTGATATTGAGCTGTATCTGTCC	59.6	204
R- GTGTTCTGTTGTATGCTTCTAATGG	59.2	
F- ATTGCTCTAACCACCATATCTCTTG	59.9	150
R- TCATGTCCTTGTAGTAGCTCTCATC	59.0	
**HN gene**		
F- TACTTCTCCCCTGCCCTACT	58.9	249
R- TACCGGGTTGAGTCTTGCTT	58.9	
F- CGGAAGTCTTGCAGTGTGAG	58.8	123
R- TAACCTCCCGTGTACCATCG	58.8	
F- GATTGCATCTGCACTCGGTT	58.9	154
R- GCTATTTGCAGCCCCATTGA	59.8	

Supplementary Material 2. Phylogenetic tree construction using NDV isolates based on a partial sequence of the Fusion (F) protein gene. The F gene nucleotide sequences analyzed comprised a fragment of 749 bp and involved 53 nucleotide sequences: The sequences of the four Ethiopian isolates from the present study, three strains used in vaccine production at the NVI, Ethiopia, and 46 sequences available in the GenBank database. The evolutionary history was inferred using the Neighbor-Joining algorithm and the software MEGA v7, with 1000 bootstrap values. The bootstrap values are displayed above branches. The recently circulating NDV isolates clustered under Genotype VI of the Class II of viruses. The Ethiopian isolates sequenced in this study (gray rectangles) and the NVI vaccine strains (black rectangles) are highlighted in the figure. The strains used in the preparation of the current NVI vaccines were grouped in the phylogenetically distant Genotype II, Class II. The new isolates were named: NDV/Haramaya/2013, NDV/Legetafo/2014, NDV/Addis/Ababa/2017, and NDV/Debre/zeit/2018.

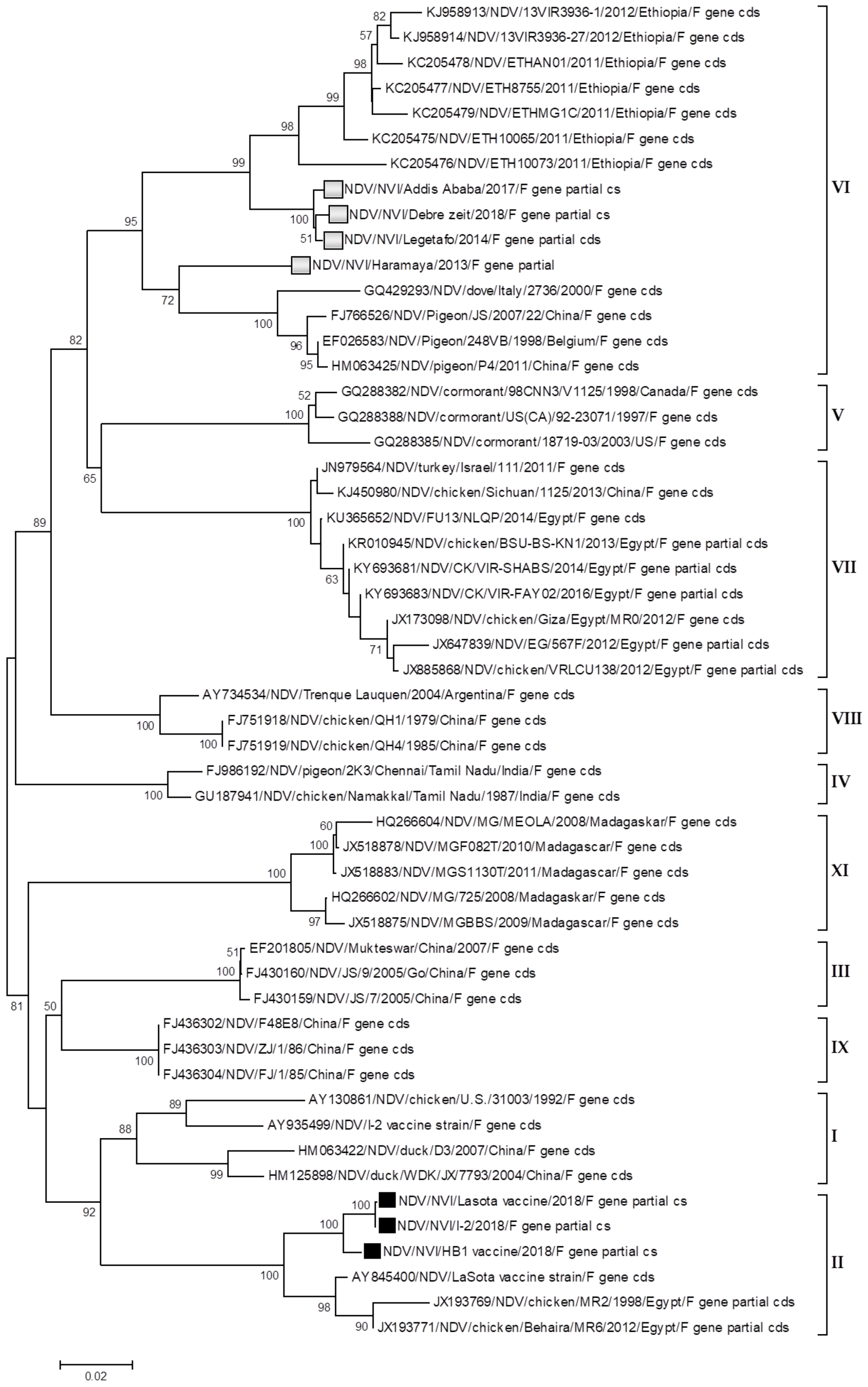

KJ958913/NDV/13VIR3936-1/2012/Ethiopia/F gene cds
KJ958914/NDV/13VIR3936-27/2012/Ethiopia/F gene cds
KC205478/NDV/ETHAN01/2011/Ethiopia/F gene cds
KC205477/NDV/ETH8755/2011/Ethiopia/F gene cds
KC205479/NDV/ETHMG1C/2011/Ethiopia/F gene cds
KC205475/NDV/ETH10065/2011/Ethiopia/F gene cds
KC205476/NDV/ETH10073/2011/Ethiopia/F gene cds
NDV/NVI/Addis Ababa/2017/F gene partial cs
NDV/NVI/Debre zeit/2018/F gene partial cs
NDV/NVI/Legetafo/2014/F gene partial cds
NDV/NVI/Haramaya/2013/F gene partial
GQ429293/NDV/dove/Italy/2736/2000/F gene cds
FJ766526/NDV/Pigeon/JS/2007/22/China/F gene cds
EF026583/NDV/Pigeon/248VB/1998/Belgium/F gene cds
HM063425/NDV/pigeon/P4/2011/China/F gene cds
VI
GQ288382/NDV/cormorant/98CNN3/V1125/1998/Canada/F gene cds
GQ288388/NDV/cormorant/US(CA)/92-23071/1997/F gene cds
GQ288385/NDV/cormorant/18719-03/2003/US/F gene cds
V
JN979564/NDV/turkey/Israel/111/2011/F gene cds
KJ450980/NDV/chicken/Sichuan/1125/2013/China/F gene cds
KU365652/NDV/FU13/NLQP/2014/Egypt/F gene cds
KR010945/NDV/chicken/BSU-BS-KN1/2013/Egypt/F gene partial cds
KY693681/NDV/CK/VIR-SHABS/2014/Egypt/F gene partial cds
KY693683/NDV/CK/VIR-FAY02/2016/Egypt/F gene partial cds
JX173098/NDV/chicken/Giza/Egypt/MR0/2012/F gene cds
JX647839/NDV/EG/567F/2012/Egypt/F gene partial cds
JX885868/NDV/chicken/VRLCU138/2012/Egypt/F gene partial cds
VII
AY734534/NDV/Trenque Lauquen/2004/Argentina/F gene cds
FJ751918/NDV/chicken/QH1/1979/China/F gene cds
FJ751919/NDV/chicken/QH4/1985/China/F gene cds
VIII
FJ986192/NDV/pigeon/2K3/Chennai/Tamil Nadu/India/F gene cds
GU187941/NDV/chicken/Namakkal/Tamil Nadu/1987/India/F gene cds
IV
HQ266604/NDV/MG/MEOLA/2008/Madagaskar/F gene cds
JX518878/NDV/MGF082T/2010/Madagascar/F gene cds
JX518883/NDV/MGS1130T/2011/Madagascar/F gene cds
HQ266602/NDV/MG/725/2008/Madagaskar/F gene cds
JX518875/NDV/MGBBS/2009/Madagascar/F gene cds
XI
EF201805/NDV/Mukteswar/China/2007/F gene cds
FJ430160/NDV/JS/9/2005/Go/China/F gene cds
FJ430159/NDV/JS/7/2005/China/F gene cds
III
FJ436302/NDV/F48E8/China/F gene cds
FJ436303/NDV/ZJ/1/86/China/F gene cds
FJ436304/NDV/FJ/1/85/China/F gene cds
IX
AY130861/NDV/chicken/U.S./31003/1992/F gene cds
AY935499/NDV/I-2 vaccine strain/F gene cds
HM063422/NDV/duck/D3/2007/China/F gene cds
HM125898/NDV/duck/WDK/JX/7793/2004/China/F gene cds
I
NDV/NVI/Lasota vaccine/2018/F gene partial cs
NDV/NVI/I-2/2018/F gene partial cs
NDV/NVI/HB1 vaccine/2018/F gene partial cs
AY845400/NDV/LaSota vaccine strain/F gene cds
JX193769/NDV/chicken/MR2/1998/Egypt/F gene partial cds
JX193771/NDV/chicken/Behaira/MR6/2012/Egypt/F gene partial cds
II
0.02

Supplementary Material 3. *In vitro* expression of the NDV recombinant F and HN antigens encoded by the adenovirus transfer vectors constructed. Western blot expression analysis of the HN (**A**) and F (**B**) antigens in HEK293SF cells transfected with the adenovirus transfer vectors pShuttle-HN-β-actin and pShuttle-F-β-actin, respectively, (lane 1, cell lysate pellets; lane 2, cell lysate supernatants). The recombinant proteins were detected with a chicken polyclonal anti-NDV at the expected molecular weight, associated to the cell lysate pellet fraction following lysis and separation by centrifugation. Panels C and D show the detection of the F and HN proteins, respectively, in HEK293SF cells, mediated by a chicken polyclonal anti-NDV (**C**) or a monoclonal anti-HN antibody (**D**). Lanes 1 and 2 in C) correspond to the expression product of transfer vectors pShuttle-F-CMV and pShuttle-F-β-actin, respectively. Lanes 1 and 2 in D) show the expression products mediated by the pShuttle-HN-CMV and pShuttle-F-HN-CMV vectors, respectively. Panel **E**) shows the detection in DF-1 avian cells of the F protein encoded by the pShuttle-F-CMV vector in lane 1, and the same protein encoded by the pShuttle-F-β-actin vectors in lane 2, detected by the polyclonal antibody. Panel **F** shows the co-expression of GFP protein mediated by the pAdTrack-HN-GFP-transfected vector in HEK293A cells, and the HN recombinant protein (lane in inner rectangle) detected by the monoclonal anti-HN antibody. By Western blot it was possible to detect the foreign antigens at the expected molecular weight of 59 kDa (for the F protein) and 63.5 kDa for the HN protein. In all cases, the antigens were detected associated to the cell lysate pellet fraction after cell lysis, centrifugation and analysis of the separated fractions.

For detection, HEK293A or DF-1 cells were seeded in 6-well plates at 0.3 × 106 cells per well in DMEM supplemented with 10% FBS. After reaching around 80% confluence, the medium was replaced with fresh DMEM without serum or antibiotics and the cultures were transfected with 1 ug of each vector using Polyethylenimine (PEI Linear, MW 25,000, PolyScience, USA) as transfection reagent and a mass ratio DNA:reagent of 1ug:3uL. Six hours later the media was removed, the cells were washed twice with PBS and fresh DMEM with serum was added. Seventy-two hours later, the cultures were harvested and assayed for NDV antigen expression by either immunodot or SDS-PAGE and Western Blot. In brief, samples were resolved by SDS-PAGE under reducing conditions and electrotransferred onto nitrocellulose membranes using a TransBlotTM transfer semi-dry (BioRad, USA). Detection of the recombinant products was achieved with a 1:200 dilution of a chicken polyclonal antibody to Newcastle disease virus (abcam, USA) followed by a goat anti-chicken IgY-HRP antibody 1:5000 (abcam, USA). Mice anti-HN monoclonal antibody (clone 9F7, abcam, USA) was also used for detection of the HN antigen, followed by a mouse anti-IgG peroxidase conjugate antibody (Sigma, USA). The nitrocellulose membrane was blocked with 5% skim milk; the primary antibody was incubated for 2 h followed by washes with PBST (PBS–0.1% Tween-20) and the secondary antibody was added for 1 h and subsequently washed. Protein bands were visualized with Clarity™ Western ECL Substrate (Bio-Rad, Canada).

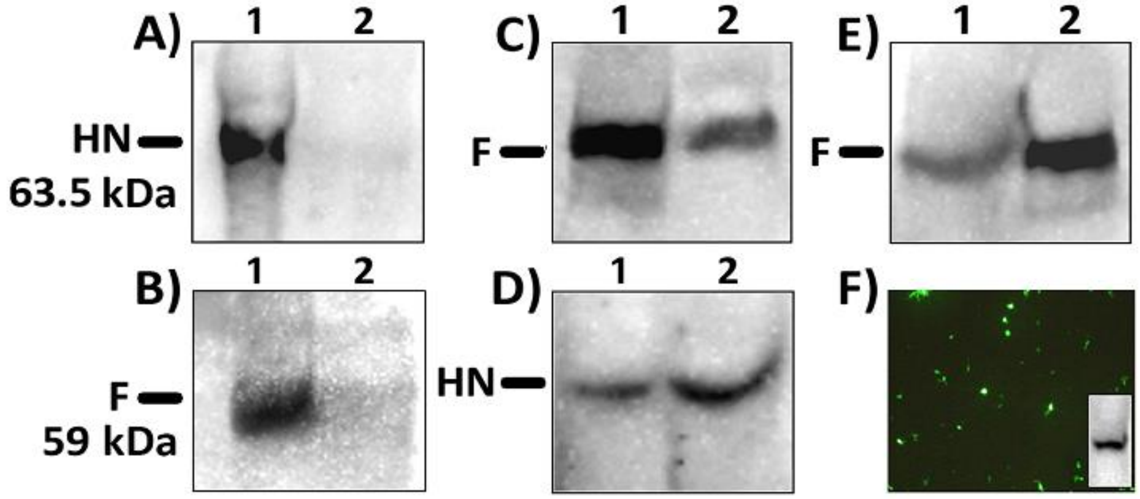

Supplement: Supplementary file 1 [file vaccines-08-00338-s001.pdf]
